# Wighteone exhibits an antitumor effect against EGFR L858R/T790M mutation non-small cell lung cancer

**DOI:** 10.7150/jca.54574

**Published:** 2021-05-05

**Authors:** Peiyuan Sun, Yana Qu, Yuna Wang, Jing Wang, Xuanjun Wang, Jun Sheng

**Affiliations:** 1 Key Laboratory of Pu-er Tea Science, Ministry of Education, Yunnan Agricultural University, Kunming, Yunnan, China.; 2 College of Science, Yunnan Agricultural University, Kunming, Yunnan, China.; 3 College of Food Science and Technology, Yunnan Agricultural University, Kunming, Yunnan, China.; 4 State Key Laboratory for Conservation and Utilization of Bio-Resources in Yunnan, Kunming, Yunnan, China.

**Keywords:** NSCLC, EGFR, L858R/T790M mutation, wighteone, antitumor effect

## Abstract

Non-small cell lung cancer (NSCLC) harboring activating EGFR mutations were initially treated by first-generation EGFR tyrosine kinase inhibitors (EGFR-TKIs), unfortunately, the efficacy of these drugs is limited, mostly frequent due to T790M mutation. Although osimertinib has been approved to treat patients with T790M-positive NSCLC, the majority of patients will develop C797S mutation and suffer diseases again. Therefore, more novel therapeutic strategies for T790M mutation-positive NSCLC are urgently required. We hypothesized that wighteone, a natural compound isolated from plant derivatives, has antitumor effects against NSCLC with T790M mutation. In this study, we created a Ba/F3 cell line harboring EGFR L858R/T790M mutation (Ba/F3 EGFR L858R/T790M cell line), and then used this cell line and a human NSCLC cell line with EGFR L858R/T790M mutation (NCI-H1975) to investigate the effects and mechanism of wighteone. The results showed that wighteone inhibited cell proliferation, suppressed EGFR signaling pathway, caused cell cycle redistribution and induced cell apoptosis. Our studies suggest that wighteone may provide a novel potential therapeutic strategy for NSCLC patients with T790M mutation.

## Introduction

Lung cancer is one of the most commonly diagnosed cancer and the leading cause of cancer-related mortality worldwide, with non-small cell lung cancer (NSCLC; comprising 85% of all lung cancers) accounting for more than 1.5 million deaths per year 1-4. Epidermal growth factor receptor (EGFR), a member of ErbB (HER) receptor tyrosine kinases (RTKs) family, plays key roles in regulation of numerous normal physiological processes, such as cell proliferation, differentiation, metabolism and apoptosis 5-7. Conversely, abnormal activation of EGFR was found in approximately 50% of NSCLC patients. Thus, targeting EGFR has provided an effective anticancer strategy, and EGFR has become a well-established critical target for the treatment of NSCLC 8-10.

EGFR tyrosine kinase inhibitors (EGFR-TKIs) have recently shown efficacies toward NSCLC, including gefitinib and erlotinib, which have been widely used to treat NSCLC with in-frame deletions of exon 19 and exon 21 L858R point activating mutation. Unfortunately, after 9-14 months of treatment, almost all patients developed drug resistance 11, 12. For more than 50% of these patients, a potential mechanism of clinically acquired drug resistance involves a second mutation of EGFR at threonine790 (T790M) 13, 14. Increasing evidence has reported that the second mutation (T790M) leads to an increased affinity between EGFR and ATP, resulting in a resistance to EGFR-TKIs 15. To overcome the resistance of T790M, third-generation TKIs are efficacious in EGFR T790M mutation-positive NSCLC, one of which, osimertinib, has been approved by the US Food and Drug Administration (FDA) 16, 17. However, after the treatment of osimertinib, the occurrence of resistance to osimertinib due to a new mutation via Cys797 to serine (C797S) will be a major obstacle. Patients who have developed the C797S mutation will undergo disease progression again 18. Therefore, due to the limited treatment options available for NSCLC, more novel therapeutic strategies are emergently required for patients with lung cancer harboring T790M mutation.

Natural products present in people's daily life, the anti-cancer activities of natural products which attracts considerable attention 19-22. Wighteone, a natural flavonoid compound, is widely distributed in natural plants, such as *Cudrania cochinchinensis, Erythrina suberosa* and* Glycyrrhiza glabra* 21, 23, 24. In recent years, wighteone has been reported to inhibit cell proliferation and induce apoptosis against cancer 25. However, the potential efficacy of wighteone against NSCLC has not been evaluated. In this study, we used Ba/F3 EGFR L858R/T790M cell line and NCI-H1975 cell line to evaluate the effect of wighteone against EGFR L858R/T790M mutant (EGFR*^L858R/T790M^*). More importantly, we investigated the mechanisms of the resulting antitumor efficacy.

## Materials and Methods

### Reagents and compounds

RPMI 1640 medium and fetal bovine serum (FBS) were obtained from Thermo Fisher Scientific (Pittsburgh, PA, United States). Penicillin-streptomycin (P/S) solution was obtained from Solarbio (Beijing, China). Neo Transfection System and Kits were obtained from Invitrogen (Carlsbad, CA, United States). Wighteone, obtained from BioBioPha Co., Ltd. (Kunming China) with purification of 99.0% by HPLC, was dissolved in dimethyl sulfoxide (DMSO; Amresco, Houston, TX, United States) and stored at -20 ºC. CellTiter 96 Aqueous One solution proliferation kit (MTS; Promega, Fitchburg, WI, United States) was purchased from Promega (Fitchburg, WI, United States). Antibodies against p-EGFR (Tyr1068), EGFR (1005), p-AKT, AKT, p44/42MAPK (Erk1/2), p-p44/42 MAPK (Erk1/2), Bax, Bcl-2, caspase3, cleaved-caspase3, caspase9, cleaved-caspase9, β-tubulin and GAPDH were purchased from Cell Signaling Technology (Beverly, MA, United States). Antibodies against PARP-1, cleaved PARP1, cyclin E, cyclin A, and CDK2 were purchased from Abcom (Lake Placid, NY, United States). Anti-mouse IgG peroxidase-linked whole antibodies and anti-rabbit IgG peroxidase-linked species-specific whole antibodies were purchased from Thermo Fisher Scientific (Waltham, MA, United States).

### Cell culture

The Ba/F3 cell line and the WEHI-3 cell line (myelomonocytic leukemia, macrophage-like, BALB/c mouse cells) as well as a human NSCLC cell line NCI-H1975 (harboring EGFR L858R/T790M mutation) were obtained from the Cell Bank in the Chinese Academy of Sciences (Kunming, China). The WEHI-3 cell line and the entire NSCLC cell lines were cultured in RPMI 1640 medium supplemented with 10% FBS and 1% P/S. The WEHI-3 cell medium was collected every two days and filtered as the source of IL-3. The Ba/F3 cell line was cultured in RPMI 1640 supplemented with 10% FBS, 1% P/S, and 1% WEHI-3 conditioned medium; the medium was changed every two days. All cells were cultured in a humidified atmosphere containing 5% CO_2_ at 37 ºC.

### Construction of stable Ba/F3 EGFR L858R/T790M cell line

Before electroporation, the cells were collected and washed with phosphate buffered saline (PBS), and then resuspended in R resuspension buffer (included with Neon Kits) at a final viable cells density of 5 × 10^7^ / mL. Then, 0.9 mL cell suspension was gently mixed with 0.1 mL sterile plasmid, and electroporation was conducted using the Neon Transfection System at a condition of 1600 V, 10 ms, 3 pulses, according to the manufacturer's instructions. After electroporation, the cells were immediately added to a plate with the appropriate volume of RPMI 1640 medium containing FBS and IL-3 and incubated at 37 ºC in a humidified 5% CO_2_ incubator for 48 h. Then, the medium was changed for IL-3-deprived medium containing FBS and P/S. When filled in the plate, the cells were moved to 96 well plates for the selection of IL-3-independent clones. After selection, cells were subjected to monoclone.

### Flow cytometry analysis

EGFR expression levels of transfected Ba/F3 cells were determined by flow cytometry. After monoclone, IL3-independent cells were collected and washed twice with PBS, then stained with fluorescein isothiocyanate (FITC)-conjugated anti-human EGFR antibody for 30 min at room temperature, and washed twice with PBS again. The samples were analyzed in BD FACS Calibur flow cytometer (BD Bioscience, San Jose, CA, United States).

### Western blot

Samples containing equal amounts of proteins (60 μg) were loaded onto sodium dodecyl sulfate polyacrylamide gel electrophoresis (SDS-PAGE) and subsequently transferred onto PVDF membranes. The membranes were blocked with TBST buffer containing 5% non-fat milk at room temperature for 1 h and then probed with indicated primary antibodies overnight at 4 ºC. After washed 3 times with TBST buffer, the membranes were incubated with the HRP-conjugated secondary antibodies for 1 h at room temperature. HRP was detected using the Prolight HRP Chemiluminescent Kit (Tiangen Biotech, Beijing, China) and FluorChem E System (ProteinSimple, Santa Clara, CA, United States).

### Cell proliferation

For IL3-independent assays, 3 × 10^4^ cells/well were seeded in 96-well plates, and allowed to grow in medium with or without IL3 for 48 h. Cell viability was measured by MTS according to the manufacturer's protocol. For the effect of wighteone on the survival cells of Ba/F3 L858R/T790M cells and human NSCLC cells NCI-H1975, 3 × 10^4^ cells/well were seeded in 96-well plates, and then treated with various concentration of wighteone (0, 0.625, 1.25, 2.5, 5, 10 or 20 μM) and EGF (20 ng/ml) for 48 h. The samples were finally measured at 490 nm using a microplate reader.

### Colony formation

6 × 10^2^ cells/well were plated in six-well plates and treated with the indicated concentration of wighteone for 24 h. The cells were then cultured for an additional 14 days, with medium changes once every 2 days. After fixation with 1 mL of formaldehyde for 5 min, the cells were stained with 1 mL of 0.1% crystal violet in the dark for 30 min. Images were captured using a camera (Canon, Tokyo, Japan).

### Cell apoptosis assay

Cell apoptosis was analyzed using an annexin V/propidiumiodide (PI) detection kit (BD Biosciences, PA, United States) and BD FACS Calibur flow cytometry. 6 × 10^5^ cells/well were plated in six-well dishes and then treated with wighteone for 24h. Then, cells were collected and incubated in 100 μL of binding buffer, and 5 μL of annexin V and 10 μL of PI were added to the suspension. After incubation for 15 min at room temperature in the dark, 400 μL 1 × binding buffer was added to each tube, and the samples were analyzed by flow cytometry within 1 h.

### Cell cycle analysis

NCI-H1975 cells (6 × 10^4^ cells/well) were seeded in six-well dishes and treated with the indicated dose of wighteone for 24 h. The cells were washed with PBS, and then fixed in 70% ethanol at 4 ºC overnight. Then, the cells were washed twice with PBS and stained in binding buffer containing 50 μg/mL of PI and 100 μg/mL of RNase A for 30 min. The samples were then analyzed by flow cytometry (BD FACS Calibur).

### Docking simulation

The 3D structure of EGFR L858R/T790M mutant (PDB code: 3W2P) was obtained from Protein Data Bank. DiscoveryStudio version 4.0 was used to the preparation of ligand and receptor. AutoDock Tools version 1.56 was used for grid and docking according to the literature. Docking parameters were set the default values, except number of GA runs was set to 20 and maximum number of evals (medium) was set to 5,000,000. The confirmations with lowest binding energy were finally selected out of 10 different conformers for each docking simulation, and then the receptor-ligand interaction was further analyzed.

### Statistical analysis

All results are expressed as the mean ± the standard error of the mean (SEM) from three or more independent replicates. The differences between two groups were statistically analyzed using either the Student's *t* test or one-way ANOVA. *p* < 0.05 was considered statistically significant. All of the statistical analyses were performed using the GraphPad Prism version 5.0 (San Diego, CA, United States).

## Results

### A stable Ba/F3 EGFR L858R/T790M cell line was created and identified

**Figure 1:**
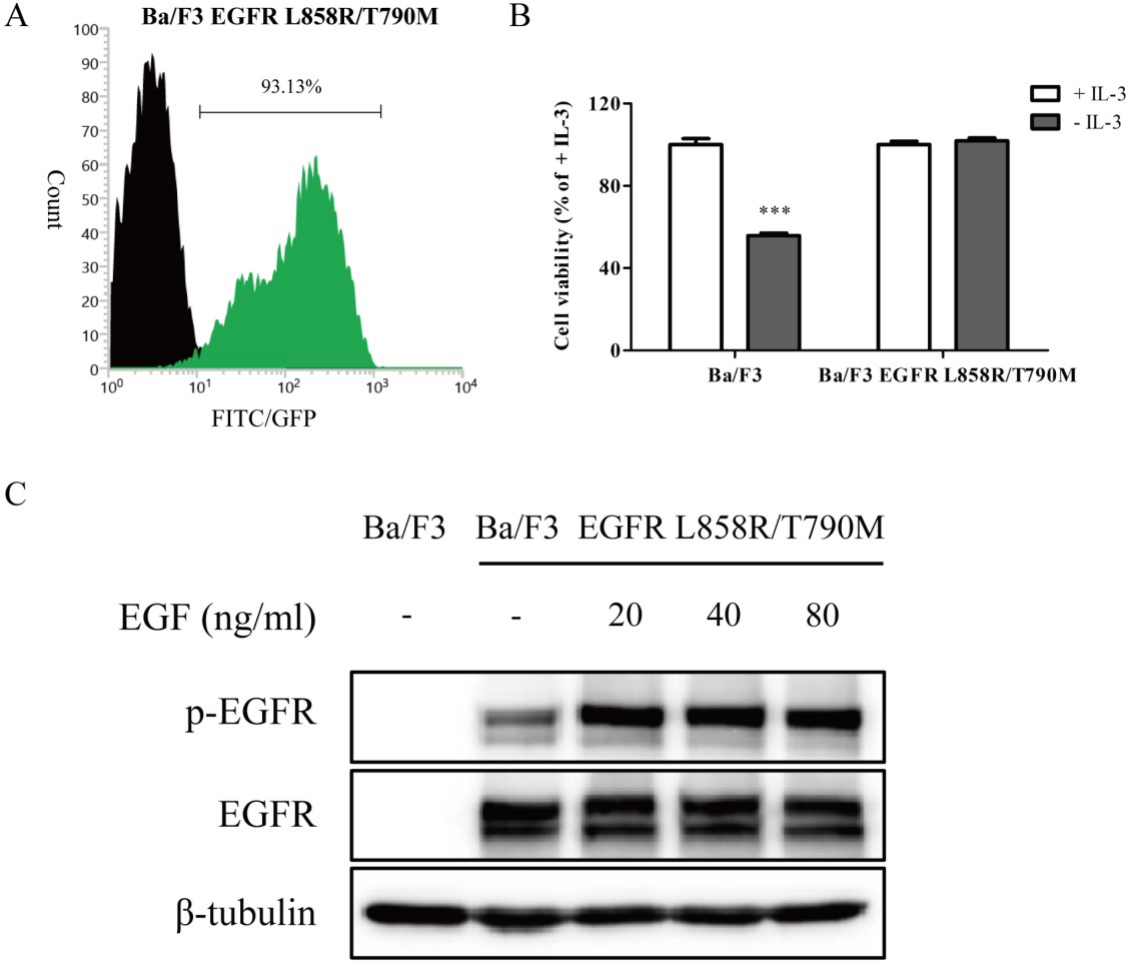
**A stable Ba/F3 EGFR L858R/T790M cell line was constructed. (A)** After electroporation and monoclone, the EGFR expression levels of transfected cells were detected by flow cytometry. Unstained cells were denoted by black region, while cells stained with specific anti-EGFR antibody were shown as green region. **(B)** IL-3-independent growth assay of Ba/F3 and Ba/F3 EGFR L858R/T790M cells was evaluated. Values for cells grown without IL-3 were normalized to the values for cells grown with IL-3. **(C)** After cells were treated with various concentration of EGF, the expression levels of p-EGFR and EGFR were determined by Western blot. Data represent the average of three independent experiments (mean ± SEM). *** *p* < 0.001 vs + IL-3.

As an initial step in evaluating the effect of wighteone *in vitro*, we created Ba/F3 cells stably expressed EGFR L858R/T790M mutation. As shown in Figure 1A, after electroporation and monoclone deprived IL-3, the EGFR expression levels were more than 90% in transfected Ba/F3 cells. To further determine whether the transfected Ba/F3 cells were independent with IL-3, we performed a growth assay. As shown in Figure 1B, the cell viability of Ba/F3 EGFR L858R/T790M cells in absence of IL-3 was near to the presence of IL-3, while the cell viability of untransfected Ba/F3 cells was significantly dependent with IL-3. The results of Western blot showed that EGFR protein was expressed in Ba/F3 EGFR L858R/T790M cells but not in untransfected Ba/F3 cells, and EGF induced EGFR phosphorylation in Ba/F3 EGFR L858R/T790M cells (Figure 1C). Taken together, the above results suggested that Ba/F3 cells stably expressed EGFR L858R/T790M mutation were generated, and the cell line may be used in subsequent experiments.

### Wighteone exhibits inhibitory effects against EGFR*^L858R/T790M^*

**Figure 2:**
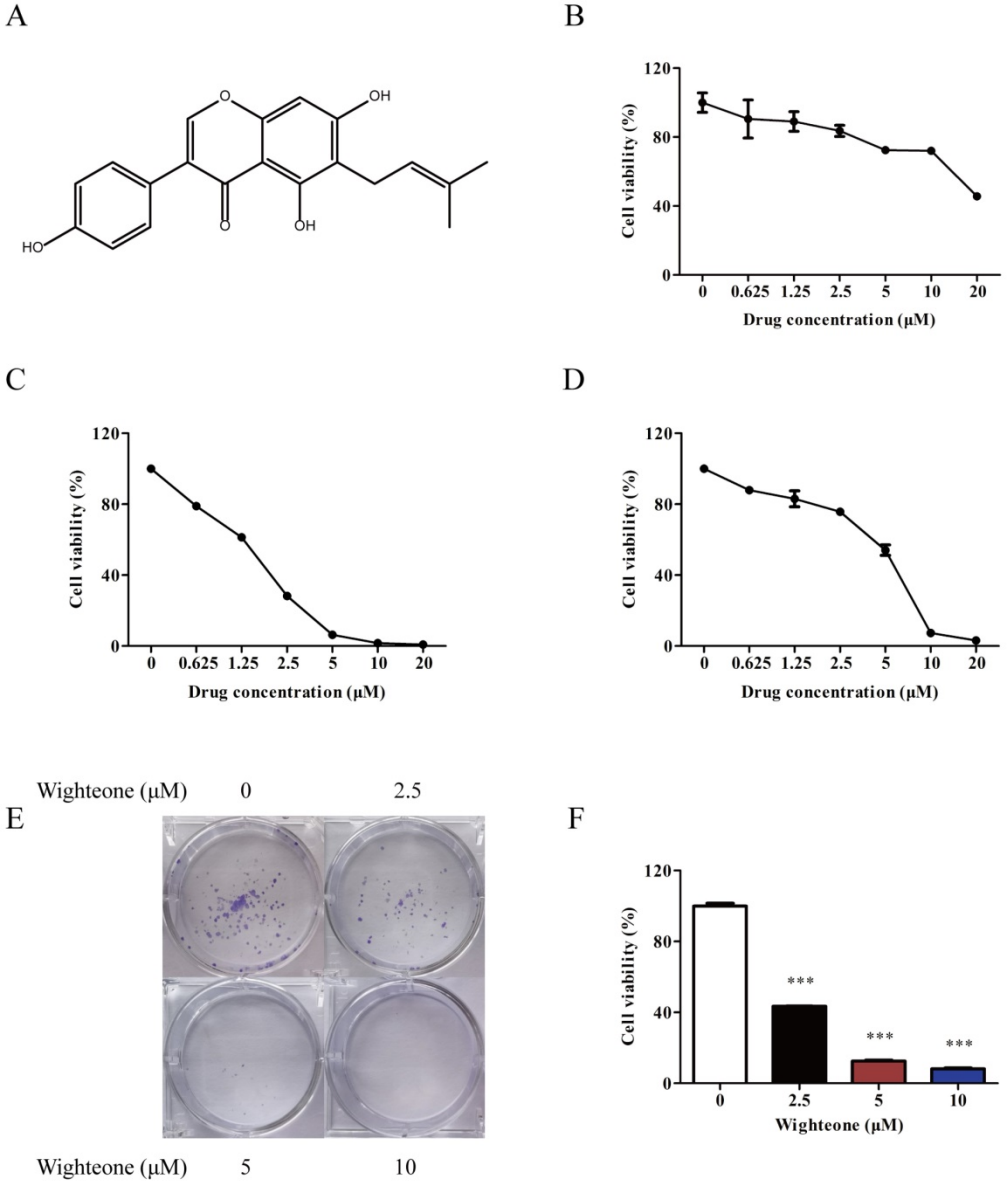
** Wighteone potently inhibited EGFR*^L858R/T790M^*. (A)** Chemical structure of wighteone. Inhibitory effects of wighteone were evaluated in Ba/F3 **(B),** Ba/F3 EGFR L858R/T790M **(C)** and NCI-H1975 **(D)** cells. **(E)** NCI-H1975 cells were treated with different concentrations of wighteone and colony efficiency was observed by a colony formation assay. **(F)** Quantitative results of clonogenic effects were analyzed. Data represent the average of three independent experiments (mean ± SEM). *** *p* < 0.001 vs the control.

To access the effect of wighteone on proliferation, we firstly tested a series of concentrations of wighteone in Ba/F3 cells, Ba/F3 EGFR L858R/T790M cells and human NSCLC cells NCI-H1975. Wighteone (Figure 2A) had a greater inhibitory effect in Ba/F3 EGFR L858R/T790M cells and NCI-H1975 cells as compared with in Ba/F3 cells (Figure 2B-D). The IC_50_ values in Ba/F3 EGFR L858R/T790M cells and NCI-H1975 cells were 1.88 and 5.70 μM, respectively. Then, we further performed a colony formation assay to determine the anti-proliferative potential of wighteone. As shown in Figure 2E, F, wighteone significantly inhibited the clonogenic potential of NCI-H1975 cells in a concentration dependent manner. These results proved the effect of wighteone on cell proliferation against EGFR*^L858R/T790M^*.

### Wighteone inhibits EGF-induced EGFR phosphorylation and its downstream signaling pathway in NCI-H1975 cells

**Figure 3:**
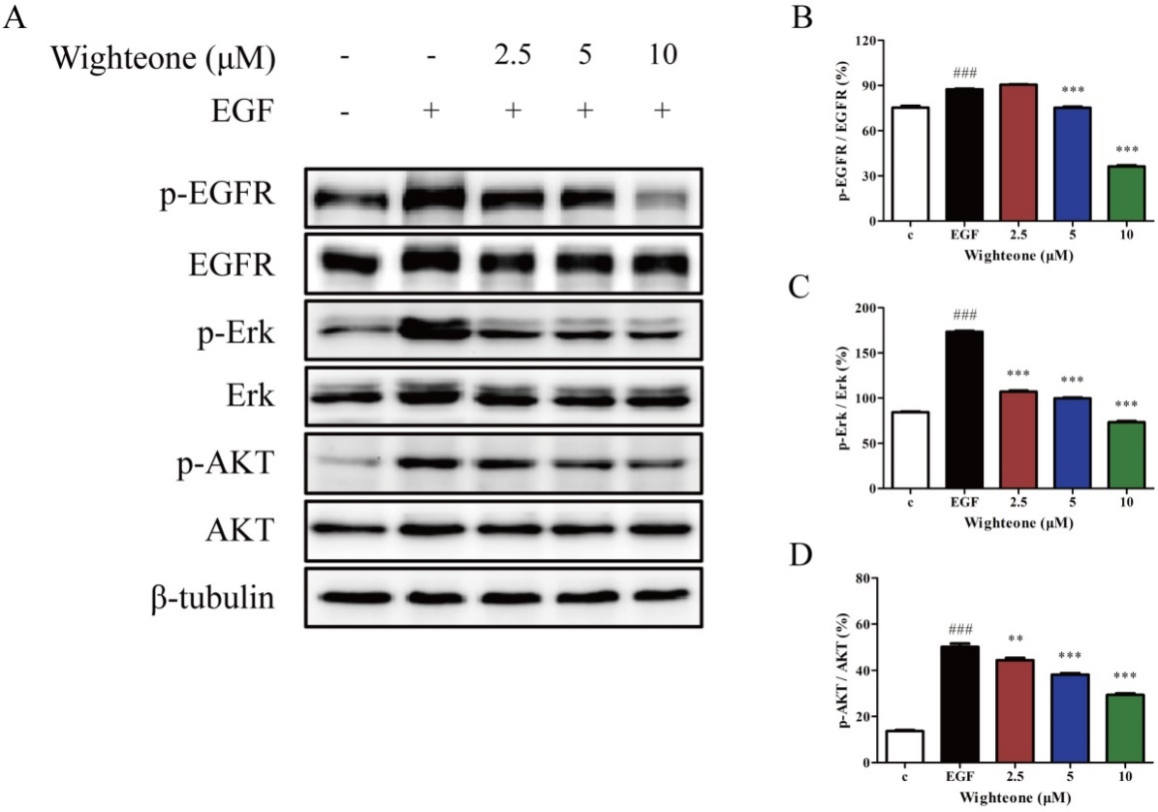
** Wighteone suppressed the EGFR signaling pathway in NCI-H1975 cells. (A)** NCI-H1975 cells were stimulated with EGF (20ng/ml) for 5 minutes and further treated with various concentrations of wighteone (2.5, 5, or 10 µM) for 16h. The expression levels of relative proteins were detected by Western blot with β-tubulin as the loading control. **(B-D)** The p-EGFR, p-Erk and p-AKT levels were quantified as percentage versus their relative total protein levels. Data represent the average of three independent experiments (mean ± SEM). ### *p* < 0.001 vs the control; ** *p* < 0.01, *** *p* < 0.001 vs EGF.

To further confirm whether the effects of wighteone might involve effects on EGFR signaling pathway, we examined the expression of several key regulators that function within the EGFR signaling pathway. As shown in Figure 3A-D, wighteone significantly suppressed EGF-induced EGFR phosphorylation as well as its downstream signaling proteins Erk and AKT in a concentration dependent manner, while the total protein levels remained unchanged in each of the groups. The results suggested that EGFR signaling pathway may play a key role in the effects of wighteone in NCI-H1975 cells.

### Wighteone induces cell cycle arrest in NCI-H1975 cells

**Figure 4:**
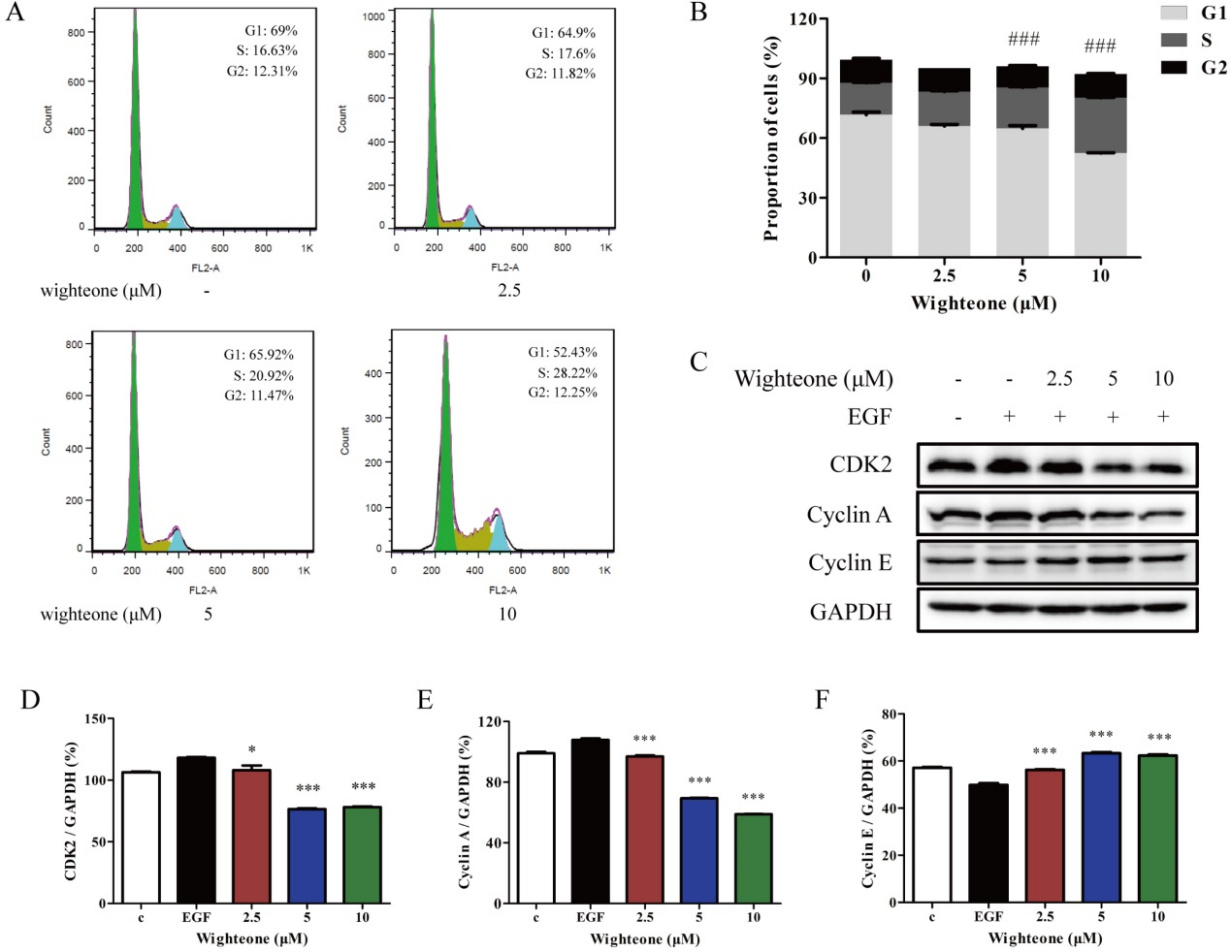
** Wighteone regulates cell cycle in NCI-H1975 cells. (A)** The cell cycle distribution (sub-G1, G0/G1, S, and G2/M) was detected by flow cytometry. NCI-H1975 cells were treated with various concentrations of wighteone for 24h. **(B)** The proportions of cells in each phase were quantified as percentages. **(C)** After NCI-H1975 cells were treated with EGF for 5 min and then further treated with wighteone for 16 h, the expression levels of CDK2, cyclin A and cyclin E were determined by Western blot, using GAPDH as the loading control. **(D-F)** CDK2, cyclin A and cyclin E protein levels were quantified. Data represent the average of three independent experiments (mean ± SEM). ### *p* < 0.001 vs the control; * p < 0.05, *** *p* < 0.001 vs EGF.

To further determine why wighteone inhibited cell growth, we investigated the cell cycle distribution. The cells were incubated with various concentrations of wighteone (2.5, 5 or 10 μM), and then subjected to propidium iodide (PI) staining and analyzed by flow cytometry. As shown in Figure 4A, B, increasing doses of wighteone dose-dependently increased the proportion of cells in the S phase. Subsequently, we further performed a Western blot assay to evaluate the effects of wighteone by examining the expression levels of relative proteins, which are the core factors in cell cycle regulation. As shown in Figure 4C-F, compared to EGF alone, wighteone significantly decreased the levels of CDK2 and cyclin A and increased the levels of cyclin E in a concentration dependent manner. Among of them, cyclin A regulates the S phases of the cell cycle along with the cofactors CDK2. Taken together, the above results revealed that the wighteone-induced down-regulation of cell viability is due to the S phase cell cycle arrest.

### Wighteone shows a potent apoptosis-inducing effect against NCI‑H1975 cells

**Figure 5:**
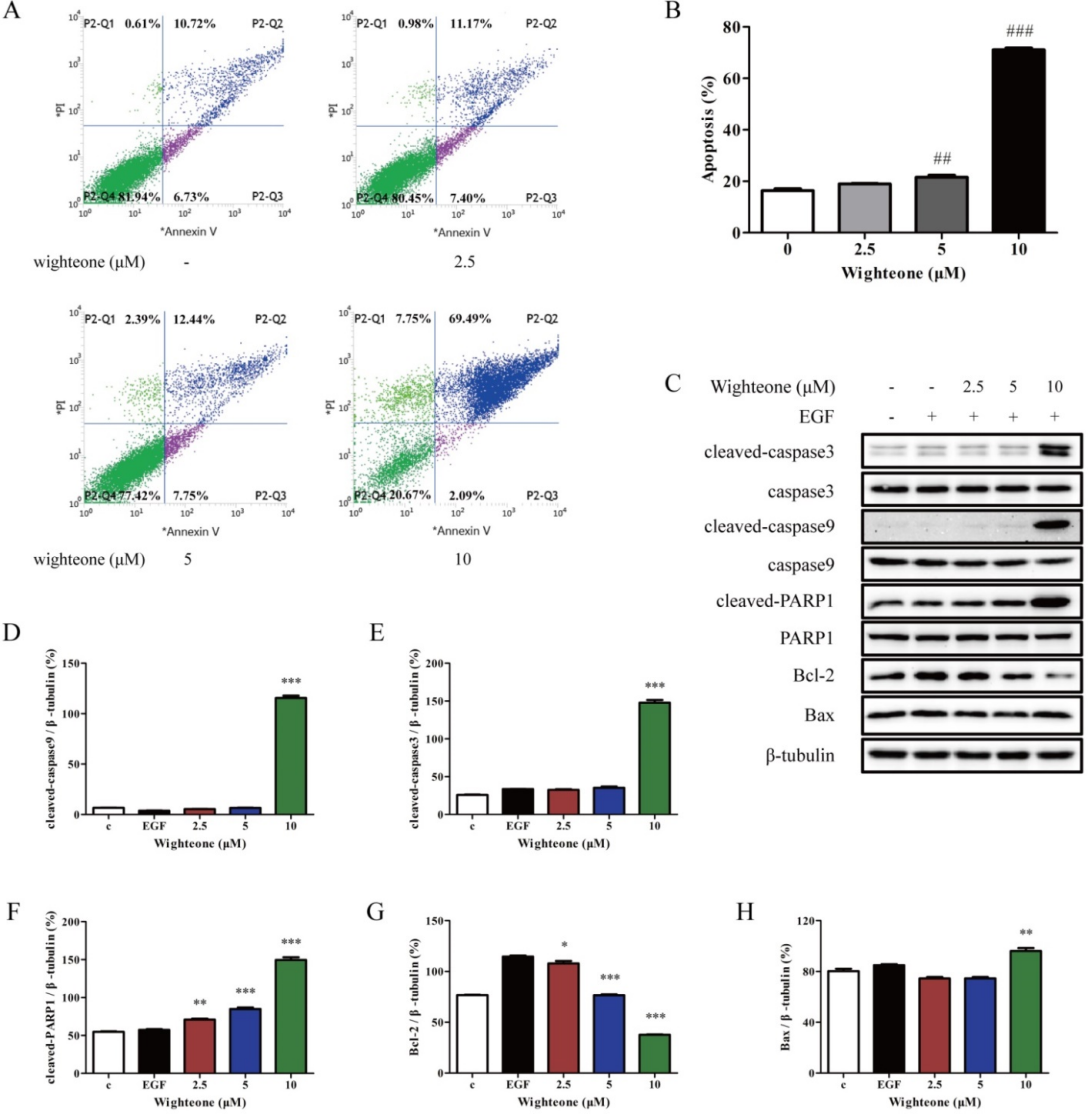
** Wighteone induces cell apoptosis in NCI-H1975 cells**. **(A)** Flow cytometry was used to detect cell apoptosis in NCI-H1975 cells treated with various concentrations of wighteone. **(B)** Quantitative results of apoptotic cells in each group were quantified as percentages. **(C)** The expression levels of relative protein were determined by Western blot, using β-tubulin as the loading control. Then, cleaved-caspase9 **(D)**, cleaved-caspase3 **(E)**, cleaved-PARP1 **(F)**, Bcl-2 **(G)** and Bax** (H)** protein levels were analyzed. Data represent the average of three independent experiments (mean ± SEM). ## *p* < 0.01, ### *p* < 0.001 vs the control; * *p* < 0.05, ** *p* < 0.01, *** *p* < 0.001 vs EGF.

To evaluate the role of wighteone in cell apoptosis of NCI-H1975 cells, we performed a flow cytometry assay where the cells were incubated with 2.5, 5 and 10 μM of wighteone for 24 h and subjected to annexin V/PI staining. As shown in Figure 5A, B, compared to the untreated control, the rates of apoptotic were significantly increased in a dose-dependent manner with respect to wighteone. In addition, we also detected the markers of cell apoptosis using Western blot and analyzed their expression levels. As shown in Figure 5C-H, the results indicated that wighteone induced a significant increase in cleaved-caspase3, cleaved-caspase9, cleaved-PARP1 and Bax and a significant decrease in Bcl-2, when compared to EGF alone.

### Wighteone directly binds to EGFR*^L858R/T790M^* in docking model

**Figure 6:**
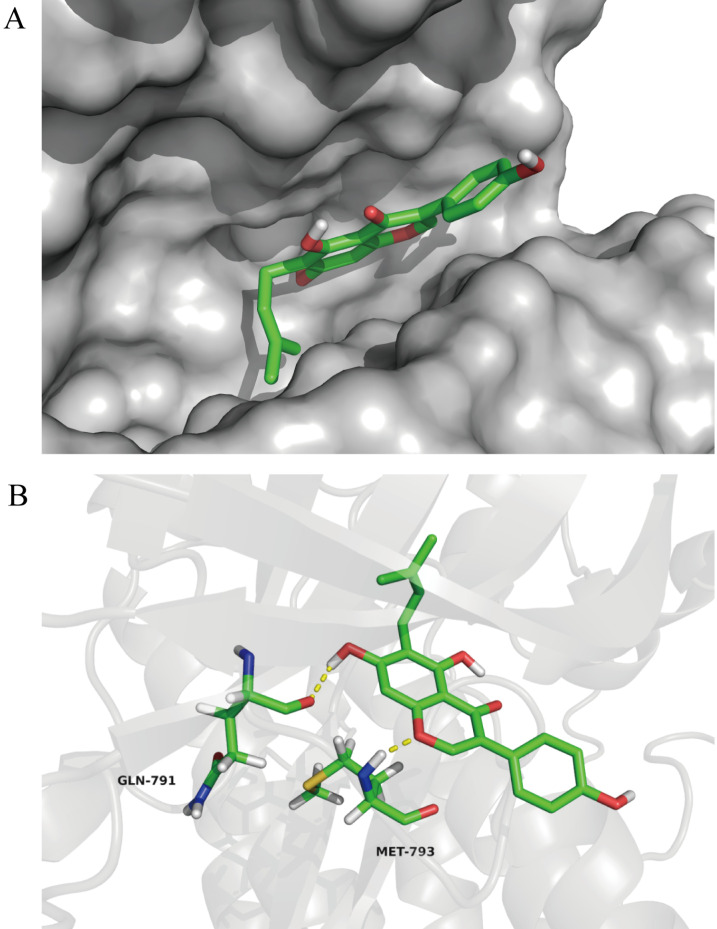
**In docking model of EGFR L858R/T790M mutant (PDB code: 3W2P) interaction with wighteone. (A)** Binding of wighteone to the ATP-binding domain of EGFR*^L858R/T790M^*. EGFR*^L858R/T790M^* was depicted by a hydrophobicity surface model (grey). **(B)** Molecular docking model of wighteone bound to EGFR*^L858R/T790M^*. Ligand and key residues are shown as sticks (C, yellow; O, red; N, blue; S, yellow; polar H, white), while hydrogen bonds are denoted by yellow dashed lines.

To further investigate the impact of wighteone for EGFR L858R/T790M mutant, we explored the possible binding modes of wighteone to EGFR*^L858R/T790M^* using the Autodock 4.2. As shown in Figure 6A, wightone is docked in the ATP-binding site of EGFR*^L858R/T790M^*. We found that wighteone forms two hydrogen bonds with carbonyl group of GLN-791 and amino group of MET-793 (Figure 6B). The above result suggested that wighteone may directly bind to EGFR*^L858R/T790M^*. A much more detailed molecular biological study elucidating this chelated binding and the mechanism of action is ongoing in our laboratory and will be reported in due course.

## Discussion

The Ba/F3 cell line is able to generate cells whose survival depends on mutant EGFR in absence of IL-3 26, 27. Based on that, we selected the cells to create the stable transfected Ba/F3 EGFR L858R/T790M cell line in this study, which was used to directly evaluate the effect of wighteone against EGFR L858R/T790M mutation. NCI-H1975 is a patient-derived NSCLC cell line harboring EGFR L858R/T790M mutation, which was used to verify the results of inhibitory effect of wighteone on Ba/F3 L858R/T790M cells. The results indicated that wighteone significantly inhibit cell viability in a concentration manner in Ba/F3 EGFR L858R/T790M and NCI-H1975 cells, with the IC_50_ values of 1.88 and 5.70 μM, respectively.

EGFR, a cell-surface protein that binds with epidermal growth factor (EGF), plays a key role in proliferation, survival, invasion and angiogenesis during the progression of NSCLC 10, 28, 29. Usually, EGF-mediated EGFR phosphorylation induces two main downstream signaling pathways, including RAS/RAF/MEK/ERK and PI3K/AKT 30, 31. Thus, EGFR has been considered as a critical signaling molecule for the treatment of NSCLC due to its multifunctional role. In this study, we demonstrated that wighteone inhibited EGFR phosphorylation despite EGF-induced. Moreover, the inhibition of EGFR phosphorylation by wighteone led to the downregulation of ERK and AKT phosphorylation.

Recently, natural compounds have attracted increasing attention in many studies because of their potential antitumor efficacy 32-35. Natural compounds may further be modified to enhance their potential and reduce their side-effects, and, more importantly, they may be applied in the treatment of all phases of cancer 25. However, the disadvantage of use is that a therapeutic strategy using natural compounds is difficult to apply without knowing molecular mechanisms of inhibitory effects 29. Wighteone is a natural flavonoid compound distributed in many plants with antitumor effects. However, its activities against NSCLC are insufficience of evidence to support. In this study, we confirmed the potential antitumor activity of wighteone against NSCLC harboring L858R/T790M mutation and investigated the molecular mechanisms. Wighteone was found to influence cell viability and colony formation by targeting EGFR signaling pathway, affect DNA damage and induce apoptosis. A molecular docking model revealed that wighteone directly binds to the ATP-binding domain of EGFR*^L858R/T790M^*. Taken together, these findings may provide a novel possibility for the treatment of NSCLC.
